# Supplementary material for: Can high-performance work practices influence employee career competencies? There is a need for better employee outcomes in the banking industry
Source: PLoS One. 2022 Mar 8;17(3):e0264764. doi: 10.1371/journal.pone.0264764 (PMC8903270; doi:10.1371/journal.pone.0264764)
Supplement: S1 File — (DOCX) [file pone.0264764.s001.docx]

**QUESTIONNAIRE**

Kindly tick (v) the answer that best represents your views (Strongly Disagree = 1, Disagree=2, Neutral=3,Agree=4. Strongly agree=5

| **n/** | **High Performance Work Practices -** | **Strongly Disagree (1)** | **Dis**  **Agree**  **(2)** | **Neutral**  **(3)** | **Agree**  **(4)** | **Strongly Agree**  **(5)** |
| --- | --- | --- | --- | --- | --- | --- |
| **1** | I feel that the balance between my work demands and non-work activities is currently about right |  |  |  |  |  |
| **2** | I received continued training to provide good service |  |  |  |  |  |
| **3** | I receive training on how to deal with complaining customers |  |  |  |  |  |
| **4** | I feel that the balance between my work demands and non-work activities is currently about right |  |  |  |  |  |
| **5** | Long-term employee potential is emphasized |  |  |  |  |  |
|  | **Career Competence** |  |  |  |  |  |
| **6** | I can clearly see what my passions are in my work |  |  |  |  |  |
| **7** | I am familiar with my shortcomings in my work |  |  |  |  |  |
| **8** | I know a lot of people outside of my work who can help me with my career |  |  |  |  |  |
| **9** | I can clearly show others what my strengths are in my work |  |  |  |  |  |
|  | **Service quality** |  |  |  |  |  |
| 10 | Customers receive prompt service from frontline employees |  |  |  |  |  |
| 11 | Frontline employees are never too busy to respond to customers' requests |  |  |  |  |  |
| 12 | This company has the customers' best interests at heart. |  |  |  |  |  |
| 13 | Customers feel safe doing business with frontline employees. |  |  |  |  |  |
|  | **Creative performance** |  |  |  |  |  |
| **14** | I carry out my routine tasks in ways that are resourceful |  |  |  |  |  |
| **15** | I come up with new ideas for satisfying customers’ needs . |  |  |  |  |  |
| **16** | I generate and evaluate multiple alternatives for novel customers problems |  |  |  |  |  |
| **17** | I have fresh perspectives on old problems |  |  |  |  |  |
|  | **Extra-role performance** |  |  |  |  |  |
| **18** | I am willing to stand up to protect the reputation of the company. |  |  |  |  |  |
| **19** | I eager to tell outsiders good news about the company and clarify their misunderstandings |  |  |  |  |  |
| **20** | I constructive suggestions that can improve the operation of the company |  |  |  |  |  |
| **21** | I willing to assist new colleagues to adjust to work environment |  |  |  |  |  |
