# Supplementary material for: Can high-performance work practices influence employee career competencies? There is a need for better employee outcomes in the banking industry
Source: PLoS One. 2022 Mar 8;17(3):e0264764. doi: 10.1371/journal.pone.0264764 (PMC8903270; doi:10.1371/journal.pone.0264764)
Supplement: Attachment — Submitted filename: PONE-D-21-22454.docx [file pone.0264764.s002.docx]

PLOS ONE

CAN HIGH PERFORMANCE WORK PRACTICES HAVE EFFECT ON EMPLOYEE CAREER COMPETENCIES? THERE IS A NEED TO IMPROVE EMPLOYEE OUTCOMES.

--Manuscript Draft--

| Manuscript Number: | PONE-D-21-22454 |
| --- | --- |
| Article Type: | Research Article |
| Full Title: | CAN HIGH PERFORMANCE WORK PRACTICES HAVE EFFECT ON EMPLOYEE CAREER COMPETENCIES? THERE IS A NEED TO IMPROVE EMPLOYEE OUTCOMES. |
| Short Title: | CAN HIGH PERFORMANCE WORK PRACTICES HAVE EFFECT ON EMPLOYEE CAREER COMPETENCIES? |
| Corresponding Author: | Damis Feruzi Kamna, Ph.D  Cyprus International University: Uluslararasi Kibris Universitesi Nicosia, CYPRUS |
| Keywords: | - High performance work practices; Human resource management; Career competencies; Extra-role performance |
| Abstract: | This study focuses on HPWPs, employee career competencies and employee outcomes, show the importance of organisation investing in human capital can be an asset to any organisational competitiveness and growth. The study examined the the effect of high performance work practices on employee outcomes in banking system. The main aims of this study are to examine 1. the effect of high performance work practices on employee career competencies in financial organisations. 2. the mediating effect of employee career competencies on the relationship between high performance work practices and employee outcomes in bank sector. The study uses quantitative approach with total of 340 respondents from various banks in Tanzania. The data was analysed using Partial Least Squares - Structural Equation Modelling (PLS-SEM). The results of the finding indicate that high performance work practices has significantly effect on employee career competencies. Similarly, employee career competencies has significantly impact on service quality, creative performance and extra-role performance in banks. Also, employee career competency do not mediate the relationship between HPWPs and service quality. |
| Order of Authors: | Damis Feruzi Kamna, Ph.D |
|  | Shiva ILKHANIZADEH |
| Additional Information: |  |
| Question | Response |
| Financial Disclosure  Enter a financial disclosure statement that describes the sources of funding for the work included in this submission. Review the [submission guidelines](http://journals.plos.org/plosone/s/submission-guidelines#loc-financial-disclosure-statement) for detailed requirements. View published research articles from [PLOS ONE](http://journals.plos.org/plosone/) for specific examples.  This statement is required for submission and will appear in the published article if the submission is accepted. Please make sure it is accurate. | The funders had no role in study design, data collection and analysis, decision to publish, or preparation of the manuscript. |

| Unfunded studies Enter: The author(s) received no specific funding for this work. Funded studies Enter a statement with the following details: - Initials of the authors who received each award - Grant numbers awarded to each author - The full name of each funder - URL of each funder website - Did the sponsors or funders play any role in the study design, data collection and analysis, decision to publish, or preparation of the manuscript? - NO - Include this sentence at the end of your statement: The funders had no role in study design, data collection and analysis, decision to publish, or preparation of the manuscript. - YES - Specify the role(s) played. |  |
| --- | --- |
| * typeset |  |
| Competing Interests  Use the instructions below to enter a competing interest statement for this submission. On behalf of all authors, disclose any [competing interests](http://journals.plos.org/plosone/s/competing-interests) that could be perceived to bias this work—acknowledging all financial support and any other relevant financial or nonfinancial competing interests.  This statement is required for submission and will appear in the published article if the submission is accepted. Please make sure it is accurate and that any funding sources listed in your Funding Information later in the submission form are also declared in your Financial Disclosure statement.  View published research articles from [PLOS ONE](http://journals.plos.org/plosone/) for specific examples. | The author(s) received no specific funding for this work. |

| NO authors have competing interests Enter: The authors have declared that no competing interests exist. Authors with competing interests Enter competing interest details beginning with this statement: I have read the journal's policy and the authors of this manuscript have the following competing interests: [insert competing interests here] |  |
| --- | --- |
| * typeset |  |
| Ethics Statement  Enter an ethics statement for this submission. This statement is required if the study involved:   - Human participants - Human specimens or tissue - Vertebrate animals or cephalopods - Vertebrate embryos or tissues - Field research   Write "N/A" if the submission does not require an ethics statement.  General guidance is provided below. Consult the [submission guidelines](http://journals.plos.org/plosone/s/submission-guidelines#loc-guidelines-for-specific-study-types) for detailed instructions. Make sure that all information entered here is included in the Methods section of the manuscript. | "N/A" |

Format for specific study types

Human Subject Research (involving human

participants and/or tissue)

Give the name of the institutional review

board or ethics committee that approved the

study

•

Include the approval number and/or a

statement indicating approval of this

research

•

Indicate the form of consent obtained

(

written/oral) or the reason that consent was

not obtained (e.g. the data were analyzed

anonymously)

•

Animal Research (involving vertebrate

animals, embryos or tissues)

Provide the name of the Institutional Animal

Care and Use Committee (IACUC) or other

relevant ethics board that reviewed the

study protocol, and indicate whether they

approved this research or granted a formal

waiver of ethical approval

•

Include an approval number if one was

obtained

•

If the study involved

non-human primates

,

add

additional details

about animal welfare

and steps taken to ameliorate suffering

•

If anesthesia, euthanasia, or any kind of

animal sacrifice is part of the study, include

briefly which substances and/or methods

were applied

•

Field Research

Include the following details if this study

involves the collection of plant, animal, or

other materials from a natural setting:

Field permit number

•

Name of the institution or relevant body that

granted permission

•

Data Availability

Authors are required to make all data

underlying the findings described fully

available, without restriction, and from the

time of publication. PLOS allows rare

exceptions to address legal and ethical

concerns. See the

[PLOS Data Polic](http://journals.plos.org/plosone/s/data-availability)

[y](http://journals.plos.org/plosone/s/data-availability)

and

[FA](http://journals.plos.org/plosone/s/data-availability#loc-faqs-for-data-policy)

[Q](http://journals.plos.org/plosone/s/data-availability#loc-faqs-for-data-policy)

for detailed information.

Yes - all data are fully available without restriction

| A Data Availability Statement describing where the data can be found is required at submission. Your answers to this question constitute the Data Availability Statement and will be published in the article, if accepted. |  |
| --- | --- |
| Important: Stating ‘data available on request from the author’ is not sufficient. If your data are only available upon request, select ‘No’ for the first question and explain your exceptional situation in the text box. |  |
| Do the authors confirm that all data underlying the findings described in their manuscript are fully available without restriction? |  |
| Describe where the data may be found in full sentences. If you are copying our sample text, replace any instances of XXX with the appropriate details.   - If the data are held or will be held in a public repository, include URLs, accession numbers or DOIs. If this information will only be available after acceptance, indicate this by ticking the box below. For example: All XXX files   are available from the XXX database (accession number(s) XXX, XXX.).   - If the data are all contained within the manuscript and/or Supporting Information files, enter the following: All relevant data are within the manuscript and its Supporting Information files. - If neither of these applies but you are able to provide details of access elsewhere, with or without limitations, please do so. For example:   Data cannot be shared publicly because of [XXX]. Data are available from the XXX Institutional Data Access / Ethics Committee (contact via XXX) for researchers who meet the criteria for access to confidential data.  The data underlying the results presented in the study are available from (include the name of the third party | All relevant data are within the manuscript and its Supporting Information files. |

| and contact information or URL).  • This text is appropriate if the data are owned by a third party and authors do not have permission to share the data.  * typeset |  |
| --- | --- |
| Additional data availability information: |  |

Manuscript	[Click here to](https://www.editorialmanager.com/pone/download.aspx?id=29423404&guid=5f740d25-07f2-4673-a3da-e02ee8d1ed7d&scheme=1)

[access/download;Manuscript;PLOSnewEffect11_4.docx](https://www.editorialmanager.com/pone/download.aspx?id=29423404&guid=5f740d25-07f2-4673-a3da-e02ee8d1ed7d&scheme=1)

**CAN HIGH PERFORMANCE WORK PRACTICES HAVE EFFECT ON EMPLOYEE CAREER COMPETENCIES? THERE IS A NEED TO IMPROVE EMPLOYEE OUTCOMES.**

# Abstract

This study focuses on HPWPs, employee career competencies and employee outcomes, show the importance of organisation investing in human capital can be an asset to any organisational competitiveness and growth. The study examined the the effect of high performance work practices on employee outcomes in banking system. The main aims of this study are to examine 1. the effect of high performance work practices on employee career competencies in financial organisations. 2. the mediating effect of employee career competencies on the relationship between high performance work practices and employee outcomes in bank sector. The study uses quantitative approach with total of 340 respondents from various banks in Tanzania. The data was analysed using Partial Least Squares - Structural Equation Modelling (PLS-SEM). The results of the finding indicate that high performance work practices has significantly effect on employee career competencies. Similarly, employee career competencies has significantly impact on service quality, creative performance and extra-role performance in banks. Also, employee career competency do not mediate the relationship between HPWPs and service quality.

**Keywords -** High performance work practices, Human resource management, Career competencies, Extra-role performance

# Introduction

The strategic human resource management (SHRM) has researchers assessing the conceivable share of using high-performance work systems (HPWS) as a method to expand an organizations' competitive advantage [1-4]. HPWS refers to an interconnected group of HR policies and practices which involves thorough selective staffing, general training and development, incentive rewards, good work-life balance, adequate empowerment, job security and, great career opportunities which are calculated to improve employees' career competencies and achieve organizational objectives [1,5-9]. Although, preceding HPWS study has displayed a relationship with employee wellbeing and organization performance [1,10,11]. The new HPWS research are often being critiqued for its extensive management-centric viewpoint, which prioritizes management objectives over employee well being [12]. This viewpoint is apparent in its limited economic outlook of HPWS research, which centers around improving the competence of HR practices [13][14], while employees effects on HPWS has not been given the appropriate attention [15].

As a result of this, some researchers have requested for studies which are more centered on employee research and also features the managerial and functionalist viewpoints which is a well-adjusted tactic that seeks to evade the sidelining of employee outcomes in HPWS [12,16,17]. [8] also contended that the HR systems should be aimed at some organizations' tactical objectives and should function by managing employees at work, and especially, the planning and handling of their careers [18]. Debatably, human factor are the most esteemed factors in modern organizations, and offering employees a long-term secure career is a win-win position for organizations and their employees. [19] described a career as a lasting course, which comprises of a series of actions, mindsets or behaviors that happens in a person’s work life. Career development relates to the development of employees that is deemed advantageous to both the individual and organization [20], which also encompasses a structured, official and well-calculated effort to accomplish equilibrium between the individual’s career prerequisites and the organization’s work force requests. Employees must take responsibility for their employability by acquiring experience and career competencies.

For employees to maintain a job in this dynamic labour market, individuals need progressive career competencies that can help them in the course of their career [21]. When an organization provides the appropriate HPW practices, employees discover that their job competencies meet up with the organizations standards; they would likely exhibit employee outcomes such as service quality, creative performance and extra-role behaviour. No study has been able to make this conceptualization; this paper hereby proposes to develop a conceptual framework that examines High performance work practices effects on career competencies, career competencies with employee outcomes; service quality, creative performance and extra-role behaviour and finally, career competencies is examined as a mediator of the impacts on service quality, creative performance and extra-role behaviour.

# Literature reviews

## HPWPs and career competencies

This study focuses more on employee driven HPWP, which has not received enough attention. Studies relating to the effect of HPWP on employees’ pro-organizational behaviour are habitually grounded on the social exchange theory (SET). This denotes that employees feel a responsibility to respond organizations benefits, when they receive favourable treatments from organizations [22]. There are social exchange connections that spur affirmative job or organizational conclusions, when the organization cares for their employees [23]. Employees that attain economic and socio-emotional capabilities in a workspace are usually compelled to reimburse the organization in several ways. It can be said that in the absence of specific tactical goals within an organization, the HR work systems would be unable to lead the employees. It is necessary for an organization to select and design the appropriate HR practices that are not only allied with each other), but also affiliated with the organization's plan so as to accomplish the suitable organizational outcomes [1,10]. There are several components of HPWSs such as training, empowerment, work-life balance, rewards, selective staffing, job security and career opportunities [24,25]. It can be said that HPWPs are exhibited through organization’s prominence on these components. Organizations that partake in HPWPs often foster motivation amongst their employees and which results in positive outcomes for organizations [25].

There is a growing need for employees to actively manage their careers; it is gradually becoming essential for them to acquire the crucial competencies that empower them to succeed [26]. It is therefore important that they become prolific in their career-related competencies that can help them steer through their career. Career competencies concern an individual’s entire career and could be distinguished from job skills and work capabilities, which are geared towards effectively executing a job [27]. It can be said that career competencies relate to knowledge, skills and abilities. Career construction theory positions that persons build their careers by enforcing meaningful experiences to their vocational and occupational performances [28]. Employees should acquire the necessary resources to deal with career loads and pressures [29]. With HPWPs in place, employees relish working for the organization while following their objectives and building their career competencies.

Organizations’ ability to carefully recruit the appropriate personnel’s helps them acquire talented individuals, who boost their productivity. [30] logically states that empowerment encourages skillfulness and growth, which enables employees achieve their goals by overcoming hurdles. When employees continue to receive the right training their skills and abilities are boosted which gives them the chance to use their empowerment in their jobs successfully. Employees are inspired to manage their career demands effectively when they work in an environment where is adequate compensation, elevation in their careers and job security successfully [31]. Previous study done by [25] discovered that HPWPs had a significant relationship with Career adaptability. This hypothesis is hereby adopted:

*H1: HPWPs significantly influences Career competencies.*

## Career competencies on employee outcomes

Career competencies are presumed to play a vital role in upholding the employee’s worth to the organization [32]. Service quality, creative performance and extra-role performance are the 3 imperative employee outcomes of career competencies tested in this study. This study concentrates on these outcomes because no empirical study has tested them with career competencies. Previous study done tested career adaptability with met expectations, creative performance and extra-role performance [25]. Service quality is the process of delivering a service, and the value of the service outcome. It is the gap between the expectation of the service and satisfaction derived.

Service quality is a determining factor in the provision of service. It does not only become a source of identity by customers but it becomes an image of the organization. When employees have the knowledge, skills and abilities required to carry out their job, they are able to provide supreme service to their customers [33]. Creative performance the creation of “*products, ideas, or procedures that satisfy two conditions, namely, they are novel or original, and they are potentially relevant for, or useful to an organization*” [34,35]. Employees who are curious about new ways to carry out their work efficiently and effectively can successfully run their careers [28]. When employees are confident in their careers, they search for opportunities for personnel development by solving problems and gaining new skills [36].

Extra-role performances are employee work behaviors that involve providing services to customers that stretch farther than their formal role requirements [37]. Employees who posses career competencies are willing to go above and beyond the line to satisfy their customers. Organizations prefer to recruit employees who perform extra-role behavior. Due to the limited empirical evidence on these relationships, we hereby propose the following hypothesis:

*H2a: Career competency significantly influences service quality.*

*H2b: Career competency significantly influences creative performance.*

*H2c: Career competency significantly influences extra-role performance*.

Career competencies as a mediator

Organizations that operate with the HR practices motivates the employees to go the extra mile to satisfy the customers, explore new ways to carry on their responsibilities to achieve the organizations set goals, which inadvertently boosts the service quality. An organization with HPWPs incorporates employees who posses the knowledge, skills and abilities to provide extra-role and creative performances [25]. This notion gives rise to these hypotheses:

H3: Career competencies significantly influence a relationship between HPWP on (a) service quality (b) creative performance (c) extra-role performance.

Career

competencies

HPWPs

H1

service quality

extra-role

performance

creative

performance

H2c

H2b

H2a

Figure 1. Conceptual model

# Methodology

The conceptual model of this study is shown in Fig 1.

## Data collection and sample

Data from this survey were collected through online questionnaire conducted with banking employees across Tanzania - De Es salaam, Arub, Nwanza, and dodoma. We ensure that prospective respondents are eligible to participate in this survey, only main staffs with years of experience in the field were selected. Micro-finance bankings were avoided in order to improve the generalization of our findings. A purposive quantitative research employed for this study and respondents were assured their responses would be kept confidential. We contacted the supervisors and managers of each bank branches to seek their approval. In addition, 534 bank employees agreed to participate in the survey and, due to the current global situation, we mailed the questionnaires to each of them. There were 340 responses from respondents, which represents a 63.7% response rate. We deleted 6 incomplete questionnaires and 334 valid questionnaires were used for the data analysis. The sample of the employees consist of 195 (59.58%) male, and 139 (40.42%) female. With highest age between 25–34 years at 41.91% 59.8% are supervisors with mostly Bachelor Degree at 44.91%. The banking employees’ highest work experience are between 7 - 9 years at 29.94%.

Table 1

### The demographic characteristics of the sample

|  |  | **Frequen**  **cy** | **%** |
| --- | --- | --- | --- |
| **Gender** | Female | 139 | **40.42** |
|  | Male | 195 | 59.58 |
| **Age** | 18–24 years | 39 | **11.68** |
|  | 25–34 years | 140 | **41.91** |
|  | 35–44 years | 97 | **29.04** |
|  | 45 and above | 58 | **17.36** |
| **Marital Status** | Single | 30 | **8.98** |
|  | Married | 294 | **88.02** |
|  | Divorced/Widowed | 10 | **2.99** |
| **Position** | Marketing Manager | 33 | **9.88** |
|  | Accounting Manager | 110 | **59.88** |
|  | Supervisors | 145 | **43.41** |
|  | Operational Manager | 90 | **2.69** |
|  | HR Manager | 8 | **2.39** |
| **Educational level** | Primary education | 10 | **2.99** |
|  | Secondary education | 23 | **6.89** |
|  | Diploma | 115 | **34.43** |
|  | Bachelor Degree | 150 | **44.91** |
|  | Master’s Degree | 26 | **7.78** |
|  | Ph.D. | 10 | **2.99** |
| **Organization work experience** | <4 years  4 -6 | 75  68 | **22.45**  **20.36** |
|  | 7 -9 | 100 | **29.94** |
| > 9years 91 **27.24** | | | |

## Measurement Scales

High Performance Work Practices were measured using 5 items and adapted from previous literature [31]. These items focused on the key factors of High Performance Work practices that have been extensively used in many literature, these include training, empowerment, work-life balance, rewards, selective staffing, job security and career opportunities [24,25]. Employees were asked to what extent their organization uses High Performance Work Practices on a five-point Likert scale, ranging from Strongly Disagree (1) to Strongly agree (5). A reliability test using Cronbach’s coefficient was conducted. The alpha value was 0.899, indicating acceptable reliability of the construct.

Career’ competencies were measured using 4 items adapted from [27]. These items were measured on a five-point Likert scale. The employees were asked to assess their career competencies in the organisation, ranging from strongly disagree (1) to Strongly agree (5). A reliability test using Cronbach’s coefficient was conducted. The alpha value was .879, indicating acceptable reliability of the construct.

Service quality were measured using 4 items adapted from [38]. Service quality have been extensively used in many literature [39-41]. These items were measured on a five-point Likert scale. The employees were asked to quality of service received within the organisation, ranging from strongly disagree (1) to Strongly agree (5). A reliability test using Cronbach’s coefficient was conducted. The alpha value was .879, indicating acceptable reliability of the construct.

Creative performance was adapted from scales developed by [42]. 4 items were used to assess the working environment on improvisation methods to improve performance in line with previous studies [26][42]. we captured employees’ perceptions of carrying out routine tasks in ways that are resourceful. The employees were asked to respond to the six statements describing the creative performance in banking environment on a five-point Likert scale, ranging from strongly disagree (1) to Strongly agree (5). A reliability test using Cronbach’s coefficient was conducted.

Cronbach’s alpha was 0.882, indicating acceptable reliability of the construct.

Extra-role performance were measured using 4 items adapted from [43]. Extrarole performance have been extensively used in many literature [44-47]. The employees were asked to constructive suggestions that can improve the operation of the organisation, ranging from strongly disagree (1) to Strongly agree (5). A reliability test using Cronbach’s coefficient was conducted. Cronbach’s alpha was 0.869, indicating acceptable reliability of the construct. Also, we instructed the staffs of the bank to provide information on high performance work practices, career’ competencies, creative performance, service quality and extra-role performance. This ensured the study to adopt few approaches to reduce possible common source variance as suggested by [48] and we ensured that respondents' responses are anonymous and confidential.

## Data analysis

This study carried out different tests to confirm our proposed model. We tested for the analysis of descriptive statistics. The model fit and other factor measures were checked through confirmatory factor analysis (CFA), through measurement model, the internal consistency and validity were measured. We tested the full model in CFA consist of five variables and we determined the model fitness indices with adequate model fit as required by [49,50]. Also, we tested the research model fit with structural equation modeling using JASP 10.0.0.13 statistical software tool that measures relationships of direct and indirect effects, in relation to previous studies [51,52].

# Results and Findings

## Measurement Model

To test the measurement model, the measures of the data analysis must measure the items internal consistency and convergent validity. First, a preliminary test was performed, we check the eigenvalues of components extracted and the analysis provided more than one factor with a eigenvalue greater than 1, as shown in Fig. 2. The initial factor component (34%) of the total variance were explained. The eigenvalue component factors range from 1.263 to 7.146 and the percentage of total variance explained ranged from 34.8% to 71.6%. As a result, the variance of the common method does not reveal any serious problems in this study.

Furthermore, the results of skewness and kurtosis indices indicate that there is data normality of the model. As indicated that both skewness and kurtosis indices should not exceed |2.3| to check for data normality. The value of skewness and kurtosis indices are between |2.3|. Hence, the respondents data in this study indicate appropriateness for further factor analysis. In addition, Bartlett’s test of sphericity were employed to measure the adequacy of the sampling and Kaiser–Meyer–Olkin (KMO). The results shown significant statistics of χ^2^ (210) = 4018.258 and the KMO measure = 0.879 > 0.500 respectively

Figure 2. Eigenvalue verse components.

Secondly, we measured the measurement model based on the internal consistency and convergent validity. This measures the factor loadings (γ), composite reliability (CR), average variance extracted (AVE) and Cronbach’s alpha (α). As suggested [53] that the recommended values for reliability of each construct must > 0.70. Therefore, the values of Cronbach’s alpha (α) of the study ranged from 0.882 to 0.899, and values of each construct exceeds 0.7 threshold value [53,54] suggesting good internal consistency. Also, the factor loadings (γ), composite reliability (CR) and average variance extracted (AVE) were measured. The value of standard factor loading < 0.4 were deleted as recommended by [53]. Table 2 indicate that all values of factor loading are above 0.40. Also, the acceptable CR values for each construct should be > 0.70 while the AVE values should also be > 0.50 as recommended by [53]. The Cronbach's alpha and CR values exceed 0.7 threshold and AVE exceeds 0.5 threshold value. Thus, The value of standard factor loading, AVE, CR and Cronbach's alpha satisfy the suggested thresholds. As presented in Table 2.

Table 2: Summary of Exploratory analysis

| **Construct Measurement** | **Factor**  **Loading**  **(λ)** | **Skewness** | **Kurtosis** |
| --- | --- | --- | --- |
| **High Performance Work Practices**  **CR = 0.878, AVE = 0.590, α = 0.899**  **CP1** | 0.735 | -1.17 | 0.808 |
| **CP2** | 0.799 | -1.14 | 1.39 |
| **CP3** | 0.844 | -1.12 | 1.20 |
| **CP4** | 0.702 | -0.735 | 0.095 |
| **CP5** | 0.753 | -1.38 | 1.67 |

| **Career’ competencies**  **CR = 0.799, AVE = 0.500, α = 0.879** |  |  |
| --- | --- | --- |
| **CC1** | 0.718 -0.649 | -0.103 |
| **CC2** | 0.658 -1.341 | -0.434 |
| **CC3** | 0.764 -0.571 | -0.298 |
| **CC4**  **Service quality**  **CR = 0.814, AVE = 0.525, α = 0.790** | 0.683 -1.223 | -0.516 |
| **SQ1** | 0.739 0.132 | -0.820 |
| **SQ2** | 0.630 0.192 | -0.769 |
| **SQ3** | 0.811 -0.096 | -1.798 |
| **SQ4**  **Creative performance**  **CR = 0.826, AVE = 0.544, α = 0.882** | 0.706 -0.140 | -0.611 |
| **CP1** | 0.700 -0.766 | 0.198 |
| **CP2** | 0.702 -1.20 | 1.430 |
| **CP3** | 0.776 -0.469 | -0.194 |
| **CP4**  **Extra-role performance**  **CR = 0.842, AVE = 0.577, α = 0.869** | 0.768 -0.385 | -0.374 |
| **ERP1** | 0.795 -0.943 | 0.882 |
| **ERP 2** | 0.916 -0.950 | 0.546 |
| **ERP 3** | 0.710 -1.34 | 1.86 |
| **ERP4** | 0.578 -0.579 | -0.265 |

We tested the discriminant validity which measures the square root of the AVE of each construct if it is higher than the correlation between the constructs [53,55]. Accordingly, the diagonal value (in bold) of each construct is higher than the correlation between the construct in the model as shown in Table 3. Thus, the diagonal values of the square root of AVE are greater than inter-correlations among variables. This implies that no cross-factor loadings among items within the constructs. Therefore, the discriminant validity satisfy the requirement which acceptable to procedure to structural model. Also, means, standard deviations, and bivariate correlations in Table 3 shows that there is positive correlation between the variables.

### Table 3 Correlation coefficient of the constructs

| **Constructs**  **HPWPs** | **Mean**  3.822 | **SD** 0.128 | **HPWPs**  (**0.768**) | **CC** | **SQ** | **ERP** | **CP** |
| --- | --- | --- | --- | --- | --- | --- | --- |
|  |  |  |  |  |  |  |  |
| **CC** | 3.379 | 0.139 | 0.497 | (**0.707**) |  |  |  |
| **SQ** | 3.715 | 0.179 | 0.310 | 0.485 | (**0.725**) |  |  |
| **ERP** | 3.827 | 2.242 | 0.258 | 0.289 | 0.133 | (**0.759**) |  |
| **CP** 2.848  *** p < .05, ** p < .01, *** | | 0.149  **** p < .** | 0.265 **001** | 0.354 | 0.233 | 0.561 | (**0.737**) |

## The structural model

To further test the validity of the latent constructs, we used confirmatory factors analysis. The overall results of the model fit of the research model is Table 4 displayed. The chi square/degree of freedom (χ^2^/df), Goodness of Fit Index (GFI), Bentler-Bonett Non-normed Fit Index (NNFI), Normed Fit Index (NFI), Root mean square error of approximation (RMSEA), Comparative Fit Index (CFI) and Standardized root mean square residual (SRMR) values as suggested range of recommended value [56,57]. This result suggests that the research model is suitably fit.

Table 4: Model fit summary of the research model (confirmatory factor analysis)

**Fit index**	**Recommended**

**value**

**Research**

**model**

**Chi square/degree of freedom (χ**

**/df)**

**Goodness of Fit Index (GFI)**

≥ 0.80

0.897

**Bentler**

**-**

**Bonett Non**

**-**

**normed Fit Index**

≥ 0.80

0.93

9

**^2^**	≤ 3.00	2.138

## (NNFI) Normed Fit Index (NFI) 0.907 Root mean square error of approximation 0.060

| **(RMSEA)** |  |  |
| --- | --- | --- |
| **Comparative Fit Index (CFI)** | ≥ 0.90 | 0.948 |

## Tucker-Lewis Index (TLI) 0.939 Standardized root mean square residual 0.041 (SRMR)

## Hypotheses tests

The study tested the structural method using a bootstrapping procedure with 1000 bootstrap samples evaluated [58]. The results of the hypothesis tests show the relationship between the constructs for the research model as shown in Table 5 and the path analysis is presented showing the summary of results - the relationships, estimates of the original coefficients (β), Std. Error (Standard Error), z-value and level of significance. Also, the R^2^ for CC is 33.3%, which proven to be significant to HPW, as stated by [55]. Similarly, the predictive power of CC (47%) proved to be a strong predictor for factors of cognitive load, social influence, and learning motivation, and the predictive power of SQ (32.4%), ERP (10.5%) and CP (17.1%) proved to be a strong predictor of CC in strategic human resource management.

From the results, hypotheses H1, H2a, H2b and H2c were empirically supported. Thus, the relationships among these hypotheses of the current research model were found to be significant in predicting employee outcomes in organisation practices. Among these predicting factors, HPW has a significant impact of (H1: β = 0.590, p < 0.001) on employee career’s competencies. This suggested that HPWs influence employee career’s competencies in organisation practices. Also, employee career’s competencies has a significant impact of (H2a: β = 0.386, ρ < 0.001) on employee service quality in organisation practices. This suggested that employee career’s competencies is an important factor that influences employee service quality in organisation practices. In addition, employee career’s competencies has a positive effect (H2b: β = 0.288, ρ < 0.001) on creative performance in Banks. Thus, H3 is statistically significant. The result of the findings indicates that employee career’s competencies has a significant effect (H2c: β = 0.354, ρ < 0.001) on extra-role performance in organisation practices. This suggests that employee career’s competencies influences extra-role performance in organisation practices.

Also, the indirect effect of H3a shows the career competencies significantly influence a relationship between HPWP and service quality in organisation practices, the result shows unverifiable support (H3a: β = 0.088, ρ < .0001), which indicates that employee’ career competencies does not mediate the relationship between HPWP and service quality in organisation practices. In contrast, H3b shows that career competencies significantly influence a relationship between HPWP and creative performance, the result shows empirically support (β = 0.114, ρ < .0001), this indicates that career competencies partially mediates the relationship between HPWP and creative performance in organisation practices. Also, H3c is hypothesized that career competencies significantly influence a relationship between HPWP and extrarole performance in organisation practices, the result shows empirical support (β = 0.150, ρ > .0001) that career competencies partially mediates the relationship between HPWP and extra-role performance in organisation practices. Thus, the summary of the hypotheses test results are presented in Table 5.

Table 5 hypotheses test results

| **Hypothesis** | **Estimate** | **Std. Error** | **z-value** | **p-value** | **Decision** |
| --- | --- | --- | --- | --- | --- |
| **H1:HPW→ CC** | 0.590 | 0.071 | 8.360 | 0.000 | Supported |
| **H2a:CC→ SQ** | 0.386 | 0.066 | 5.875 | 0.000 | Supported |
| **H2b:CC→ ERP** | 0.288 | 0.068 | 4.214 | 0.000 | Supported |
| **H2c:CC→ CP** | 0.354 | 0.068 | 5.196 | 0.000 | Supported |
| **H3a:HPW→CC → SQ** | 0.088 | 0.054 | 1.626 | 0.104 | Not supported |
| **H3b:HPW→CC → CP** | 0.114 | 0.058 | 1.972 | 0.049 | supported |
| **H3c:HPW→CC→ ERP** | 0.150 | 0.060 | 2.500 | 0.012 | Supported |

Supported at **p < 0.05; z > 1.96

# Discussion and Implications

The aim of this study examined why HPWs affect employee’ career competencies. We also investigate whether employee’ career competencies influence employee service quality, creative performance and whether this helps in employee’s extra-role performance in banking sectors.

First, there are some interesting findings about the effect of HPWs on employee career competencies. In-line with existing studies, the findings of this study provide an empirical support for the direct relationship between HPWSs and employee’ career competencies [24,25]. The role of HPWs tends to promote importance of careerrelated competencies in areas such as knowledge, skills and abilities through training, empowerment, and career opportunities. Accordingly, most organisations’ investing on HPWs help staff’s knowledge-intensive, set of skills and competencies in order to help development the relationships between HPWs–employee career competencies and improve organizational core capabilities.

Second, this study’s results demonstrated a significant relationship between career-related competencies and service quality. These results show that perceived employee career competencies has positive impact on organizational service quality, this implies that the employee professionals’ roles and functions is determined by professional job activities being performed within the organisation. It is a function of how qualify the employee in performing certain functions and roles. Thereby link to the employee competence on critical activities depends on level of knowledge, skills and attitude as it positively influences organisational service quality [33].

Third, the results of this study revealed that there is a significant effect between employee career competencies and creative performance which is in-line with study by [25,36]. This shows that employees are will to accomplish career goals, and feel accomplish through selective staffing, training, and rewards in order to motivate their employees’ creative performance along other social activities that might influence their creative performance. Therefore, career competencies help employee to feel more secure and protected [59].

This result of the findings which is in line with previous studies on career-related competencies and extra - role performance which further establish the relationship between career-related competencies and extra - role performance are significant to organisational practices [25,47]. Inasmuch-as the management is providing novel ideas to improve organisational outcomes when investing on HPWPs. Then employees tend to enjoy extra-role performance as long as it is befitting their future career-related competencies, in the long run. Thus, most employees tend to use their ability for the development of the organisation. Accordingly, when their organisational motivation for the employees, employees find means of achieving their career goals with actions, and new opportunities, they show loyalty by their extra-role performance capability.

Finally, our study confirmed the mediating effect of career competencies between HPWPs and creative performance and the findings of result indicate career competencies is partially mediated. This result of the findings further indicate that a strong career competencies strengthened the effect of HPWPs on organisational creative performance. That is, organization with HPWPs provide employees with creative performance grow and develop the organisation. However, the indirect relationship between HPWPs and service quality do not mediate by career competency which suggest no effect of employee career competence on the relationship between HPWPs and service quality. The result implies that employees who focus on career competency do not strengthen organizational policies but only offers employees a long-term secure career for personal factor.

This study confirmed the mediating effect of career competencies between HPWPs and extra-role performance, the findings of result indicate that career competency partially mediate the relationship between HPWPs and extra-role performance. This results of the finding further indicate that a strong career competencies strengthened the effect of HPWPs on employee extra-role performance. This result shows that, proper HPWPs help employees to perform extra function and role at any level or position in the organisation in fact beyond organisation requirements. Thus, career competence with high level of personal resources always accomplish their exceptional goal in the organisational development [60].

## Theoretical Implications

From theoretical implication, this study contributes to organisation practices in the following ways. First, we highlighted many studies on the effect of HPWSs on the employee career competence and subsequent effect of employee career competence on employee outcomes - service quality, creative performance and extra-role performance. Thus, this study provides theoretical approach to understand the mediating effect of career competency on the relationship between HPWSs and employee outcomes. This is important because no previous research has proposed model to support the argument. This empirical study shows how HPWSs utilize its effect on employee career competence. In strategic human resource management studies, service quality, creative performance and extra-role performance are imperative employee outcomes of career competencies. Thus, the analyses of these factors show a strong contribution towards HR development in banking sector.

In general HPWPs are effective in enhancing employee career competence because they serve as organisational means to employees outcomes. Also, many researchers’ pay more attention toward the HPWSs being the antecedent on different mediators tin order to measure its affect employee and organizational outcomes [25,31]. Therefore, this study extends study on the outcome of HPWSs by analyzing the boundary effect of employee career competence on the indirect relationship with employee outcome. Based on the result of employee outcomes, the implication of indirect effect of service quality, need attention of researchers on measures to improve on organisation/employee serve quality.

## Practical Implications

Practical implication of this study shows that investing in human capital is an asset to any organisational competitiveness. As well improving on human resource, meaning that organizations need to maintain and retain their resources. Satisfied employees are more likely to stay with good staffing, general training and development, incentive rewards, good work-life balance, adequate empowerment, job security and, great career opportunities as it determines employee well-being and organization performance [8,9]. Therefore, the results of the finding implicate that organizations can achieve employee outcomes by implementing proper HPW practices that help develop and manager the interrelationship among employees in any organisation.

Also, bank managers and supervisors need to focus on HPWS practice as a means of developing employee careers competency because without human capital development the organisation might be affected both internal and external. This is important because employee careers competency promotes positive environment in every organisation and, consequently, improve employee outcomes. In retrospect, managers’ should provide incentives, royalty, and performance activity so that there will be an effective means of communication, knowledge sharing, and goal target within the organisation.

More so, the findings of this study suggest that managers should develop more elusive career competence programs that will be beneficial from organisational application of high-performance work practices in workplaces. In this respect, bank managers and supervisors need to improve on employees career competences in order to promote strategic human resource management and to help reduce incompetence within organisations.

## Limitations and suggestions for future research

This study presents many limitations and we suggest for better future research. First, this study used a single-time cross-sectional approach with many advantages and significant contributions, to provide vital induction towards the study. However, longitudinal approach could be used for future study to provide a tracking trends and changes over time with the same respondents.

Secondly, the present study focuses specifically on banks in Tanzania with respective to HR policies and practices in their organisations. Thus, there is a need to extend study population, multiple location and other non-financial institutions might provide a better concept for future research. The motive of this study is measure the effect of high performance work practices on employee career competencies on services quality, creative performance and extra role performances. However, the study does not consider other HPWSs such as training, empowerment, work-life balance, rewards, selective staffing, job security and career opportunities to provide an insightful meaning to future research.

Finally, the current study looks at how high performance work practices influence employee career competencies, as well as its influences services quality, creative performance and extra role performances as a resultant. Future research should consider other non-financial institutions management and policies that help in decision making and organisational growth. Also, inclusion of other control variables - moderators e.g. Age and gender differences.

## References

1 Becker BE, Huselid MA, Becker BE, Huselid MA. High performance work systems and firm performance: A synthesis of research and managerial implications. InResearch in personnel and human resource management 1998.
2 Huselid MA. The impact of human resource management practices on turnover, productivity, and corporate financial performance. Academy of management journal. 1995;38(3):635-72.
3 Bae J, Lawler JJ. Organizational and HRM strategies in Korea: Impact on firm performance in an emerging economy. Academy of management journal. 2000;43(3):502-17.
4 Selden SC, Sowa JE. Voluntary turnover in nonprofit human service organizations: The impact of high performance work practices. Human Service Organizations: Management, Leadership & Governance. 2015;39(3):182-207.
5 Wright PM, Snell SA. Toward an integrative view of strategic human resource management. Human resource management review. 1991;1(3):203-25.
6 Way SA. High performance work systems and intermediate indicators of firm performance within the US small business sector. Journal of management. 2002;28(6):765-85.
7 Datta DK, Guthrie JP, Wright PM. Human resource management and labor productivity: does industry matter?. Academy of management Journal. 2005 Feb 1;48(1):135-45.
8 Lepak DP, Liao H, Chung Y, Harden EE. A conceptual review of human resource management systems in strategic human resource management research.

Research in personnel and human resources management. 2006; 25:217–271

9 Zhang M, Zhu CJ, Dowling PJ, Bartram T. Exploring the effects of highperformance work systems (HPWS) on the work-related well-being of Chinese hospital employees. The International Journal of Human Resource Management. 2013;24(16):3196-212.
10 Delery JE, Doty DH. Modes of theorizing in strategic human resource management: Tests of universalistic, contingency, and configurational performance predictions. Academy of management Journal. 1996;39(4):802-35.
11 Chaudhuri K. A Discussion on HPWS Perception and Employee Behavior. Global Business & Management Research. 2009;1(2):27–42.
12 Boselie P, Dietz G, Boon C. Commonalities and contradictions in HRM and performance research. Human resource management journal. 2005;15(3):67-94.
13 Godard J. A critical assessment of the high performance paradigm. British journal of industrial relations. 2004;42(2):349-78.
14 Kalmi P, Kauhanen A. Workplace innovations and employee outcomes: Evidence from Finland. Industrial Relations: A Journal of Economy and Society. 2008;47(3):430-59.
15 Macky K, Boxall P. High-involvement work processes, work intensification and employee well-being: A study of New Zealand worker experiences. Asia Pacific Journal of Human Resources. 2008;46(1):38-55.
16 Delbridge R, Keenoy T. Beyond managerialism?. The international journal of human resource management. 2010;21(6):799-817.
17 Paauwe J. HRM and performance: Achievements, methodological issues and prospects. Journal of Management studies. 2009; 46(1):129–142
18 Baruch Y. Transforming careers: from linear to multidirectional career paths:

organizational and individual perspectives. Career development international. 2004; 9(1):58–73

19 Hall DT. Associates. Career development in organization. 1st ed. San Francisco: Jossey-Bass Publishers, 1986
20 Adekola B. Career planning and career management as correlates for career development and job satisfaction. A case study of Nigerian Bank Employees.“. Australian Journal of business and management research. 2011;1(2):100–112.
21 Heijde CM, Van Der Heijden BI. A competence based and multidimensional operationalization and measurement of employability. Human Resource

Management: Published in Cooperation with the School of Business

Administration, The University of Michigan and in alliance with the Society of Human Resources Management. 2006;45(3):449-76

22 Xu T, Lv Z. HPWS and unethical pro-organizational behavior: A moderated mediation model. Journal of Managerial Psychology. 2018;33(3):265–278.
23 Cropanzano R, Mitchell MS. Social exchange theory: An interdisciplinary review. Journal of management. 2005;31(6):874-900.
24 Karatepe OM. High-performance work practices and hotel employee performance: The mediation of work engagement. International Journal of Hospitality Management. 2013;32:132-40.
25 Safavi HP, Karatepe OM. High-performance work practices and hotel employee outcomes: The mediating role of career adaptability. International Journal of Contemporary Hospitality Management. 2018;30(2):1112–1133.
26 Akkermans J, Tims M. Crafting your career: How career competencies relate to career success via job crafting. Applied Psychology. 2017;66(1):168-95.
27 Akkermans J, Brenninkmeijer V, Huibers M, Blonk RW. Competencies for the contemporary career: Development and preliminary validation of the career competencies questionnaire. Journal of Career Development. 2013

Jun;40(3):245-67.

28 Savickas ML. The theory and practice of career construction. Career development and counseling: Putting theory and research to work. 2005;1:42-70.
29 Chong S, Leong FT. Antecedents of career adaptability in strategic career management. Journal of career assessment. 2017;25(2):268-80..
30 Kong H, Wang S, Fu X. Meeting career expectation: can it enhance job satisfaction of Generation Y?. International Journal of Contemporary Hospitality Management. 2015; 27(1):147–168.
31 Karatepe OM, Vatankhah S. The effects of high-performance work practices and job embeddedness on flight attendants' performance outcomes. Journal of Air Transport Management. 2014;37:27-35.
32 Arthur MB, Rousseau DM, editors. The boundaryless career: A new employment principle for a new organizational era. Oxford University Press on Demand; 2001.
33 Lin YC, Lin ML, Chen YC. How tour guides’ professional competencies influence on service quality of tour guiding and tourist satisfaction: An exploratory research. International Journal of Human Resource Studies. 2017;7(1):1-9.
34 Oldham GR, Cummings A. Employee creativity: Personal and contextual factors at work. Academy of management journal. 1996;39(3):607-34.
35 Malik MA, Butt AN, Choi JN. Rewards and employee creative performance: Moderating effects of creative self efficacy, reward importance, and locus of control. Journal of Organizational Behavior. 2015;36(1):59-74
36 Savickas ML, Porfeli EJ. Career Adapt-Abilities Scale: Construction, reliability, and measurement equivalence across 13 countries. Journal of vocational behavior. 2012 Jun 1;80(3):661-73.
37 Bettencourt LA, Brown SW, MacKenzie SB. Customer-oriented boundaryspanning behaviors: Test of a social exchange model of antecedents. Journal of retailing. 2005;81(2):141-57.
38 Parasuraman A, Berry L, Zeithaml V. Refinement and reassessment of the SERVQUAL scale. Journal of retailing. 2002;67(4):114.
39 Janahi MA, Al Mubarak MM. The impact of customer service quality on customer satisfaction in Islamic banking. Journal of Islamic Marketing. 2017;8(4): 595-604
40 Ali M, Raza SA. Service quality perception and customer satisfaction in Islamic banks of Pakistan: the modified SERVQUAL model. Total Quality Management & Business Excellence. 2017;28(5-6):559-77.
41 Kasiri LA, Cheng KT, Sambasivan M, Sidin SM. Integration of standardization and customization: Impact on service quality, customer satisfaction, and loyalty. Journal of Retailing and Consumer Services. 2017;35:91-7.
42 Ozturk A, Karatepe OM. Frontline hotel employees’ psychological capital, trust in organization, and their effects on nonattendance intentions, absenteeism, and creative performance. Journal of Hospitality Marketing & Management. 2019;28(2):217-39.
43 Tjosvold D, Hui C, Yu Z. Conflict management and task reflexivity for team in role and extra role performance in China. International Journal of Conflict Management. 2003; 14(2):141-163
44 Chen Z, Eisenberger R, Johnson KM, Sucharski IL, Aselage J. Perceived organizational support and extra-role performance: which leads to which?. The Journal of social psychology. 2009;149(1):119-24.
45 Ahmad I, Donia MB, Khan A, Waris M. Do as I say and do as I do? The mediating role of psychological contract fulfillment in the relationship between ethical leadership and employee extra-role performance. Personnel review. 2019 48(1):98-117
46 Gupta N, Sharma V. Relationship between leader member exchange (LMX), high-involvement HRP and employee resilience on extra-role performance: Mediating role of employee engagement. Journal of Indian Business Research. 2018; 10(2):126-150.
47 Weer CH, Greenhaus JH. Managers’ assessments of employees’ organizational career growth opportunities: The role of extra-role performance, work engagement, and perceived organizational commitment. Journal of Career Development. 2020;47(3):280-95.
48 Podsakoff PM, MacKenzie SB, Lee JY, Podsakoff NP. Common method biases in behavioral research: a critical review of the literature and recommended remedies. Journal of applied psychology. 2003;88(5):879.
49 Bentler PM. Comparative fit indexes in structural models. Psychological bulletin. 1990;107(2):238.
50 Hair JF, Gabriel M, Patel V. AMOS covariance-based structural equation modeling (CB-SEM): Guidelines on its application as a marketing research tool. Brazilian Journal of Marketing. 2014;13(2).
51 Halter CP. Exploring Statistical Analysis Using JASP. Independently published; 2018.
52 Han H, Park J, Thoma SJ. Why do we need to employ Bayesian statistics and how can we employ it in studies of moral education?: With practical guidelines to use JASP for educators and researchers. Journal of Moral Education. 2018;47(4):519-37.
53 Hair JF, Anderson RE, Babin BJ, Black WC. Multivariate data analysis: A global perspective (Vol. 7).
54 Fornell C, Larcker DF. Evaluating structural equation models with unobservable variables and measurement error. Journal of marketing research. 1981;18(1):3950.
55 Chin WW. The partial least squares approach to structural equation modeling. Modern methods for business research. 1998;295(2):295-336.
56 Shi D, Maydeu-Olivares A, DiStefano C. The relationship between the standardized root mean square residual and model misspecification in factor analysis models. Multivariate Behavioral Research. 2018;53(5):676-94.
57 Chen FF. Sensitivity of goodness of fit indexes to lack of measurement invariance. Structural equation modeling: a multidisciplinary journal. 2007;14(3):464-504..
58 Lee WC, Rodgers JL. Bootstrapping correlation coefficients using univariate and bivariate sampling. Psychological Methods. 1998;3(1):91..
59 Paais M, Pattiruhu JR. Effect of motivation, leadership, and organizational culture on satisfaction and employee performance. The Journal of Asian Finance, Economics, and Business. 2020;7(8):577-88.
60 Safavi HP, Bouzari M. The association of psychological capital, career adaptability and career competency among hotel frontline employees. Tourism management perspectives. 2019;30:65-74..
