# Supplementary material for: Can high-performance work practices influence employee career competencies? There is a need for better employee outcomes in the banking industry
Source: PLoS One. 2022 Mar 8;17(3):e0264764. doi: 10.1371/journal.pone.0264764 (PMC8903270; doi:10.1371/journal.pone.0264764)
Supplement: Attachment — Submitted filename: ACADEMIC EDITOR.docx [file pone.0264764.s003.docx]

**Dear EIC/reviewers**

**We will like to appreciate your efforts in reviewing this manuscript.**

**Thank you for your comments and contributions, it helps improve the readability of the manuscript.**

**Thanks.**

- **Line 43: “[8] also contended…” should be changed by “Lepack et al. [8] also contended…”**

*Thanks for the comment*

*We have changed it to Lepack et al. [8] also stated*

- **Analogously, in line 48: “Szabó-Bálint [19] described a career…”**

*Thanks for the comment*

*We have changed it to Szabó-Bálint [19] described a career*

- **Line 101: “According to [31]…” should be changed by “According to Kong et al. [31]…”**

*Thanks for the comment*

*We have changed it to According to Kong et al. [31],*

- **Lines 107-108: “As previously studied by [26] and…” should be changed by “As previously studied by Oliinyk [26] and…”**

*Thanks for the comment*

*We has been changed to Oliinyk [26] stated*

- **Line 170: What sample calculation criteria were used?**

*Thanks for the comment*

Yes, we used Yamane [38] sample size calculator was used to establish the minimum sample size for the survey, and the result shows that 301 participants are required for the survey.

- Line 199: “… adapted from [28]” should be changed by “… adapted from Akkermans et al. [28]”. Idem in lines 204, 210 and 218

*Thanks for the comment*

*We have corrected it*

- Line 212: Change “we” by “We”.

*Thanks for the comment -* This study tested for the analysis of descriptive statistics

- Line 227: Change “… as suggested by [49]” by “… as suggested by Weer and Greenhaus [49]”

*Thanks for the comment- We have corrected it*

This ensured that this study adopted an approach to reduce common bias, as suggested by Weer and Greenhaus [49].

- All paragraphs in the "Measurement scales" section are constructed in the same way. It is not necessary to indicate 5 times “five-point Likert scale, ranging from strongly disagree (1) to strongly agree (5)". Once is enough since it is the same in all items. Rewrite this section combining concepts and not repeating the wording scheme.

*Thanks for the comment- We have corrected it*

*High-performance work practices* were measured using seven items adapted from previous literature [32]. The items focused on the key factors of high-performance work practices that have been extensively used in many kinds of literature, including training, empowerment, work-life balance, rewards, selective staffing, job security, and career opportunities [24,25]. Employees were asked about the extent to which their organizations used high-performance work practices. The reliability test showed that the Cronbach’s alpha value was 0.899, indicating acceptable reliability of the construct. *Career competencies* were measured using four items adapted from Akkermans et al. [28]. Employees were asked to assess their career competencies in the organization. Cronbach’s alpha was .879, indicates acceptable reliability of the construct. *Service quality* was measured using four items, adapted from Bettencourt et al. [39]. Service quality has been studied extensively [40-42]. The items include the quality of the service received within the organization. The overall reliability value was .879, indicating good reliability of the construct.

*Creative performance* was adapted from the scale developed by Ali and Razi [42]. In line with previous studies, Four items were used to assess the working environment, in line with previous studies [27,43]. These items capture employees’ perceptions of routine tasks in several ways. A reliability test using Cronbach’s coefficient was conducted. Cronbach’s alpha was 0.882, indicating acceptable reliability of the construct. *Extra-role performance* was measured and adapted from Ozturk and Karatepe [44]. The extra-role performance has been extensively studied [45-48]. Employees were asked to make constructive suggestions to improve the organization's operations. The Cronbach’s coefficient was 0.869, indicating acceptable reliability of the construct. The questionnaire was measured on a five-point Likert scale, ranging from strongly disagree (1) to strongly agree (5). We instructed bank employees to provide information on their high-performance work practices, career’ competencies, creative performance, service quality, and extra-role performance. This ensured that this study adopted an approach to reduce common bias, as suggested by Weer and Greenhaus [49]. We ensured that respondents' responses were anonymous and confidential.

- Revise line 236: “… as required by [50,51]”
- Rewrite sentence in line 243.
- Revise sentence in line 245-246.
- Revise the present-past in lines 248-249.

*Thanks for the comments- We have corrected it*

- This study carried out different tests to confirm the study proposed model. First, the model fit and other measurement factors were tested using CFA (Confirmatory Factor Analysis) as suggested by Podsakoff et al. [50] and Bentler [51]. Furthermore, we tested the research model relationships with Covariance-Based Structural Equation Modelling (CB-SEM) using the JASP 10.0.0.13 statistical software tool that measures relationships of direct and indirect effects of variables [52,53].
- PLS-SEM do not requires data be normal distribution. Therefore, the analysis of normality has no sense in this study (lines 251-261).

*Thanks for the comment- We have corrected it - we used Covariance Based Structural Equation Modelling (CB-SEM).*

- RRevise line 270: “… as recommended by [54]”

while [54] recommended that the AVE values should also be > 0.50.

- Line 275. “As presented in Table 2” is an incomplete sentence.

Table 2 presents a summary of the exploratory analyses (AVE, CR, Cronbach's alpha, skewness and kurtosis).

- Line 307: Revise “as stated by [55]”

*Thanks for the comment- We have corrected this section*

We conducted a correlation analysis to evaluate the relationships between the constructs [55]. Accordingly, the results show a positive and significant relationship between the constructs
